# Resurgence of respiratory syncytial virus with dominance of RSV-B during the 2022–2023 season

**DOI:** 10.3389/fmicb.2024.1376389

**Published:** 2024-04-02

**Authors:** Neli Korsun, Ivelina Trifonova, Iveta Madzharova, Ivaylo Alexiev, Iordanka Uzunova, Ivan Ivanov, Petar Velikov, Tatiana Tcherveniakova, Iva Christova

**Affiliations:** ^1^National Laboratory “Influenza and ARI”, Department of Virology, National Center of Infectious and Parasitic Diseases, Sofia, Bulgaria; ^2^Faculty of Medicine, Sofia University, Sofia, Bulgaria; ^3^Department of Infectious Diseases, Medical University, Sofia, Bulgaria

**Keywords:** respiratory syncytial virus, molecular epidemiology, genetic variability, genotype, evolution, whole-genome sequencing

## Abstract

**Background:**

Respiratory syncytial virus (RSV) is a common cause of upper and lower respiratory tract infections. This study aimed to explore the prevalence of respiratory syncytial virus (RSV) and other respiratory viruses in Bulgaria, characterize the genetic diversity of RSV strains, and perform amino acid sequence analyses of RSV surface and internal proteins.

**Methods:**

Clinical and epidemiological data and nasopharyngeal swabs were prospectively collected from patients with acute respiratory infections between October 2020 and May 2023. Real-time PCR for 13 respiratory viruses, whole-genome sequencing, phylogenetic, and amino acid analyses were performed.

**Results:**

This study included three epidemic seasons (2020–2021, 2021–2022, and 2022–2023) from week 40 of the previous year to week 20 of the following year. Of the 3,047 patients examined, 1,813 (59.5%) tested positive for at least one viral respiratory pathogen. RSV was the second most detected virus (10.9%) after SARS-CoV-2 (22%). Coinfections between RSV and other respiratory viruses were detected in 68 cases, including 14 with SARS-CoV-2. After two seasons of low circulation, RSV activity increased significantly during the 2022–2023 season. The detection rates of RSV were 3.2, 6.6, and 13.7% in the first, second, and third seasons, respectively. RSV was the most common virus found in children under 5 years old with bronchiolitis (40%) and pneumonia (24.5%). RSV-B drove the 2022–2023 epidemic. Phylogenetic analysis indicated that the sequenced RSV-B strains belonged to the GB5.0.5a and GB5.0.6a genotypes. Amino acid substitutions in the surface and internal proteins, including the F protein antigenic sites were identified compared to the BA prototype strain.

**Conclusion:**

This study revealed a strong resurgence of RSV in the autumn of 2022 after the lifting of anti-COVID-19 measures, the leading role of RSV as a causative agent of serious respiratory illnesses in early childhood, and relatively low genetic diversity in circulating RSV strains.

## 1 Introduction

Viral acute respiratory infections (ARIs) are a leading cause of infectious morbidity and mortality and have serious health, economic, and social consequences. Among the known respiratory viruses (more than 200), respiratory syncytial virus (RSV) is one of the most common etiological agents of upper and lower respiratory tract infections (LRTIs), with the greatest disease burden in infants, young children, the elderly, and immunocompromised adults. It is also a leading cause of serious LRTI (bronchiolitis and pneumonia) in children aged < 5 years. According to epidemiological data from 2019, 33 million episodes of acute LRTI, 3.6 million hospital admissions, and 101,400 death cases in children aged 0–60 months have been attributed to RSV (Li et al., 2022). RSV belongs to the family *Pneumoviridae* and the genus *Orthopneumovirus* (Lefkowitz et al., 2018). The genome of this virus is a non-segmented, single-stranded, negative-sense RNA approximately 15.2 kb in length. It encodes 11 proteins, namely NS1, NS2, N, P, M, SH, G, F, M2-1, M2-2, and L, each with specific functions (Collins and Karron, 2013). The surface glycoproteins G and F play crucial roles in the initial stage of viral infection. The G protein is accountable for attaching the virus to host cells. On the other hand, the F protein makes it possible for the viral ribonucleoprotein to enter the cell’s cytoplasm as a result of the fusion of the viral envelope with the host cell membrane (McLellan et al., 2013). During the fusion process, RSV F undergoes dramatic pre-fusion-to-post-fusion conformational changes. The active pre-fusion conformation includes six major antigenic sites (Ø and I–V) and produces more potent neutralizing antibodies. Only four sites (I, II, III, and IV) are present in the inactive post-fusion conformation (Chang et al., 2022). The F protein is the major viral antigen and is relatively conserved, whereas the G protein contains two hypervariable regions (HVRs) in the superficial ectodomain and exhibits greater genetic diversity. The highly variable C-terminal region of the G gene (HVR2) is commonly used in molecular, epidemiological, and evolutionary studies (Gimferrer et al., 2019; Sáez-López et al., 2019; Kim et al., 2023). The G and F proteins are heavily glycosylated with N-linked and O-linked sugars, which limit antibody access to the antigenic еpitopes, thereby facilitating RSV evasion from pre-existing immunity (Holmes, 2013).

RSV has a single serotype divided into two major subgroups, RSV-A and RSV-B, each comprising multiple genotypes (Mufson et al., 1985). Based on the genetic variability of the G gene, RSV-A is divided into 14 genotypes (GA1–GA7, SAA1, NA1–NA4, CB-A, and ON1), whereas RSV-B comprises 37 genotypes (GB1–GB6, GB12, GB13, SAB1–SAB4, URU1, URU2, CB1, THB, BA1–BA14, BA-C, BA-CCA, BA-CCB, JAB1, NZB1, and NZB2; Gaymard et al., 2018; Muñoz-Escalante et al., 2021). Goya et al. (2020) proposed a new classification in which the number of genotypes was reduced to three for RSV-A (GA1–GA3) and seven for RSV-B (GB1–GB7), with two additional levels of classification defined as sub-genotypes and lineages (Goya et al., 2020). Variations in the temporal and geographical distributions of individual RSV genotypes and the periodic replacement of predominant genotypes were observed (Esposito et al., 2015; Sáez-López et al., 2019; Kamau et al., 2020; Lee et al., 2021; Tabatabai et al., 2022; Kim et al., 2023). Viruses of different genotypes can co-circulate during a single epidemic within the same community. Genotyping of circulating RSV is essential for monitoring the emergence and clinical burden of new genotypes and for developing therapeutic and preventive strategies. Furthermore, analyzing the amino acid composition of RSV surface glycoproteins enables tracing of the evolutionary dynamics of the pathogen and understanding the molecular mechanisms underlying its genetic and antigenic variability.

During the COVID-19 pandemic, various public health and social distancing measures (e.g., mandatory mask-wearing, working from home, school closures, limited social gatherings, travel restrictions, quarantine, and patient isolation) were implemented to prevent the spread of SARS-CoV-2; these measures resulted in greatly reduced transmission of RSV and other seasonal respiratory viruses (Agca et al., 2021; Groves et al., 2021). After two seasons of low circulation, in the winter of 2022–2023, RSV activity returned to levels similar to those in the seasons preceding the pandemic (Jia et al., 2022; Guo et al., 2023; Pierangeli et al., 2023). In Bulgaria, RSV has been detected using real-time RT-PCR since 2010 within the national influenza surveillance system. This study aimed to explore the circulation patterns of RSV and other respiratory viruses during the COVID-19 pandemic, describe the epidemiological and clinical characteristics of RSV infection and the genetic diversity of RSV strains in the first RSV post-pandemic season, and perform an amino acid sequence analysis of the RSV surface and internal proteins.

## 2 Methods

### 2.1 Patients and specimen collection

The present study was conducted from October 2020 to May 2023, covering three epidemic seasons (2020–2021, 2021–2022, and 2022–2023) from week 40 of the previous year to week 20 of the following year. A total of 3,047 patients from different regions of the country who were ambulatory-treated or hospitalized for ARIs were enrolled in the National Influenza Surveillance Program. ARIs were defined according to the European Center for Disease Prevention and Control case definition.^1^ The study population consisted of patients of all age ranges: 728 in the first season, 482 in the second, 1,597 in the third season, and 240 inter seasons. In the first two seasons, the number of patients examined was significantly smaller due to the disruption of the national surveillance system caused by the COVID-19 pandemic. Nasopharyngeal and oropharyngeal swab specimens were prospectively collected from enrolled patients and inserted into a container containing 2 mL of virus transport medium. Specimens were obtained during the visit to the doctor or within the first 24 h of admission, within 7 days of the onset of respiratory symptoms. After collection, specimens were stored at 2°C–8°C for up to 24 h at the health facilities and then sent in ice packs to the National Laboratory “Influenza and ARI” for the detection of viral respiratory pathogens. Specimens were processed immediately, and aliquots of the primary samples were stored at −80°C.

### 2.2 Molecular detection of respiratory viruses

Viral nucleic acids were extracted automatically from 400 μL of each respiratory specimen with an elution volume of 100 μL using a commercial ExiPrep Dx Viral DNA/RNA kit and ExiPrep 16DX instrument (Bioneer, Daejeon, Republic of Korea), according to the manufacturer’s instructions. The SARS-CoV-2 and influenza A/B viruses were simultaneously screened using the FluSC2 Multiplex Real-Time RT-PCR Kit provided by the International Reagent Resource (IRR; United States) (CDC, 2022). Real-time RT-PCR was performed to subtype influenza A viruses and determine the influenza B genetic lineage using the SuperScript III Platinum One-Step qRT-PCR kit (Invitrogen, Thermo Fisher Scientific, Waltham, MA, USA), with specific primers and probes provided by IRR (USA). Amplification was performed using a CFX96 thermal cycler (Bio-Rad Laboratories, Inc., Singapore), according to the protocol recommended by CDC-Atlanta (United States; Shu et al., 2011).

The presence of eight common non-influenza respiratory viruses, namely RSV, human metapneumovirus (hMPV), parainfluenza virus (PIV) types 1/2/3, rhinovirus (RV), adenovirus (AdV), and bocavirus (BoV), was screened using multiplex real-time PCR assays with primers and probes, as previously described (Kodani et al., 2022). Three PCR mixtures were developed, including the SuperScript III Platinum One-Step qRT-PCR kit and combinations of primers and TaqMan probes labeled with different fluorescent dyes: Mixture 1: AdV + RSV + PIV1; Mixture 2: BoV + RV + PIV2; and Mixture 3: hMPV + PIV3. Positive and negative controls were included in each experiment. The RNAase-P gene was used as an internal positive control during specimen extraction. For influenza type A and B viruses, positive controls were provided by IRR (United States), while for other viruses, AmpliRun DNA/RNA Amplification Controls (Vircell, Spain) were used. Samples with a cycle threshold (Ct) value <38 were considered positive. To distinguish RSV-A and RSV-B, two sets of oligonucleotides targeting the RSV F and N genes were used for multiplex real-time RT-PCR, as described previously (Zlateva et al., 2007). The primer and probe sequences and thermocycling conditions are listed in Supplementary Table 1.

### 2.3 RSV whole-genome sequencing

Fifty-two randomly selected RSV-B-positive samples with a Ct value <30 were subjected to whole-genome sequencing. The Respiratory Virus Panel Illumina RNA Prep with Enrichment (L) Tag (Illumina, San Diego, CA, United States) and the Illumina MiSeq system with the reagent kit v.3150 cycles (Illumina, San Diego, CA, United States) were used to perform whole-genome sequencing of 40 common respiratory viruses.^2^ Before sequencing, the quality of the DNA pool libraries was verified by QIAxcel Advanced capillary electrophoresis (QIAGEN, Switzerland). Normalization of libraries was performed with a Qubit 4 Fluorometer (Thermo Fisher Scientific, Waltham, MA, United States) and Invitrogen™ Quant-iT™ 1X High Sensitivity (HS) Broad Range (BR) dsDNA Assay Kit (Invitrogen, Thermo Fisher Scientific, Waltham, MA, United States). For genome analysis, the Explify RPIP Data Analysis software (v2.0.0), available on the BaseSpace platform (Illumina, Cambridge, United Kingdom), was used. Complete or almost complete RSV genomes were obtained from 47 RSV-B-positive samples with coverage greater than 99%, mainly from samples with higher viral loads. The RSV-B sequences reported in this study have been deposited in the EpiRSV database of the GISAID under the accession numbers listed in Supplementary Table 2.

### 2.4 Phylogenetic analysis

For phylogenetic analysis, the RSV-B sequences obtained in this study were aligned with published sequences representing known genotypes and sequences of recently circulating RSV strains from different geographical regions retrieved from GenBank and the EpiRSV of GISAID. Geneious Prime® 2020.1.2. software^3^ was used for alignment and phylogenetic tree construction using the following algorithms: Tamura–Nei genetic distance model, maximum likelihood build method, and 1,000 replicates bootstrap. The final tree was visualized and annotated using iTOL software.^4^ Pairwise nucleotide distances (*p*-distances) were calculated using Geneious Prime software to compare the differences within and between genotypes. The phylogenetic tree consisted of 47 nucleotide sequences from this study, 46 reference sequences, and other RSV-B sequences available in GenBank and GISAID databases.

### 2.5 Deduced amino acid sequence analysis and glycosylation prediction

Translation of the nucleotide code into the amino acid code was performed using BioEdit software (version 7.2). To identify amino acid substitutions in G, F, and other proteins, the studied sequences were aligned and compared with the sequence of the NH118 strain (accession number MF185752), one of the first BA strains with a completely published genome.

NetNGlyc 1.0 web server^5^ was used to predict putative N-glycosylation sites with a threshold value of 0.5. To identify potential O-glycosylation sites, we used the NetOGlyc 4.0 web server, which can be accessed at https://services.healthtech.dtu.dk/service.php?NetOGlyc-4.0. N-linked glycosylation occurs when the amino acid sequence has N–X–S/T (sequon), where X is any amino acid except proline. O-glycosylation occurs when the amino acid sequence has serine and threonine configuration.

### 2.6 Statistics

GraphPad Prism version 6.0 was used for statistical analyses. Categorical data were presented as frequencies and percentages and compared using Chi-square (χ^2^) and Fisher’s exact tests. Continuous data were expressed as either means or medians, depending on the specific context. A *p-*value of less than 0.05 was considered statistically significant.

## 3 Results

### 3.1 Patient characteristics

Of the 3,047 patients examined, 71.2% (2170) were admitted to the hospital as inpatients, while 28.8% (877) were treated as outpatients. The patient’s age range was from 3 days to 91 years, with a median age of 5. Out of all study participants, 505 (16.6%) were <11 months old, 260 (8.5%) were 11–23 months old, 265 (8.7%) were 24–35 months old, 378 (12.4%) were 36–59 months old, 883 (12.4%) were 5–14 years old, 136 (4.5%) were 15–29 years old, 98 (3.2%) were 30–64 years old, 455 (14.9%) were ≥ 65 years old, and 67 (2.2%) were of unknown age. Among the study subjects whose sex was known, there were 1,587 males and 1,428 females, resulting in a male-to-female ratio of 1.11.

### 3.2 Virus detection

Viral respiratory pathogens were identified in 1,813 (59.5%) of the 3,047 patients examined. At least one viral respiratory agent was detected in 433 (49.4%) outpatients and 1,380 (63.5%) inpatients (*p* < 0.05). Single infections were proven in 1,651 (54.2%) patients, 155 (5.1%) patients were co-infected with two viruses, and seven (0.2%) were co-infected with three viruses. A total of 669 (22%) patients were positive for SARS-CoV-2, and 469 (15.4%) were infected with influenza viruses: A(H1N1)pdm09 (223, 7.3%), A(H3N2; 155, 5.1%), and B/Victoria lineages (91, 3%). Non-influenza respiratory viruses, including RSV (331, 10.9%), hMPV (59, 1.9%), PIV-1 (8, 0.3%), PIV-2 (6, 0.2%), PIV-3 (43, 1.4%), RV (199, 6.5%), AdV (106, 3.5%), and BoV (107, 3.5%), were detected in 859 (28.2%) patients (Table 1). RSV was the most prevalent seasonal non-influenza respiratory virus, followed by RV (*p* < 0.05). PIV and hMPV were identified as having the lowest frequency. The detection rates of RSV were 3.2%, 6.6%, and 13.7% during the first, second, and third seasons, respectively. The incidence rates of RSV infection among outpatients and inpatients were 10.8 and 10.9%, respectively. Approximately 20.5% (68/331) of the RSV-positive patients were co-infected with other respiratory viruses. The most common co-infecting virus was RV (23 cases, 6.9%), followed by SARS-CoV-2 (14 cases, 4.2%), BoV (13 cases, 3.9%), AdV (9 cases, 2.7%), and influenza A(H1N1)pdm09 (8 cases, 2.4%). Single cases of mixed RSV infections were found with hMPV, PIV-1, PIV-3, influenza A(H3N2), and B/Victoria lineage. Among the study participants with RSV co-infections, 55 (80.9%) were inpatients.

**Table 1:** Detected respiratory viruses among outpatients and inpatients.

	Total tested	Total positive, *n* (%)	Influenza viruses, *n* (%)			SARS-CoV-2, *n* (%)	Seasonal non-influenza respiratory viruses, *n* (%)							
			A(H1N1) pdm09	A(H3N2)	B/Vic		RSV	hMPV	PIV-1	PIV-2	PIV-3	RV	AdV	BoV
Outpatients	877	433 (49.4)	79 (9.0)	65 (7.4)	33 (3.8)	45 (5.1)	95 (10.8)	21 (2.4)	1 (0.1)	1 (0.1)	6 (0.7)	69 (7.9)	28 (3.2)	26 (3)
Inpatients	2,170	1,380 (63.6)	144 (6.6)	90 (4.1)	58 (2.7)	624 (28.8)	236 (10.9)	38 (1.8)	7 (0.3)	5 (0.2)	37 (1.7)	130 (6)	78 (3.6)	81 (3.7)
Total	3,047	1,813 (59.5)	223 (7.3)	155 (5.1)	91 (3)	669 (22)	331 (10.9)	59 (1.9)	8 (0.3)	6 (0.2)	43 (1.4)	199 (6.5)	106 (3.5)	107 (3.5)

**Figure 1:**
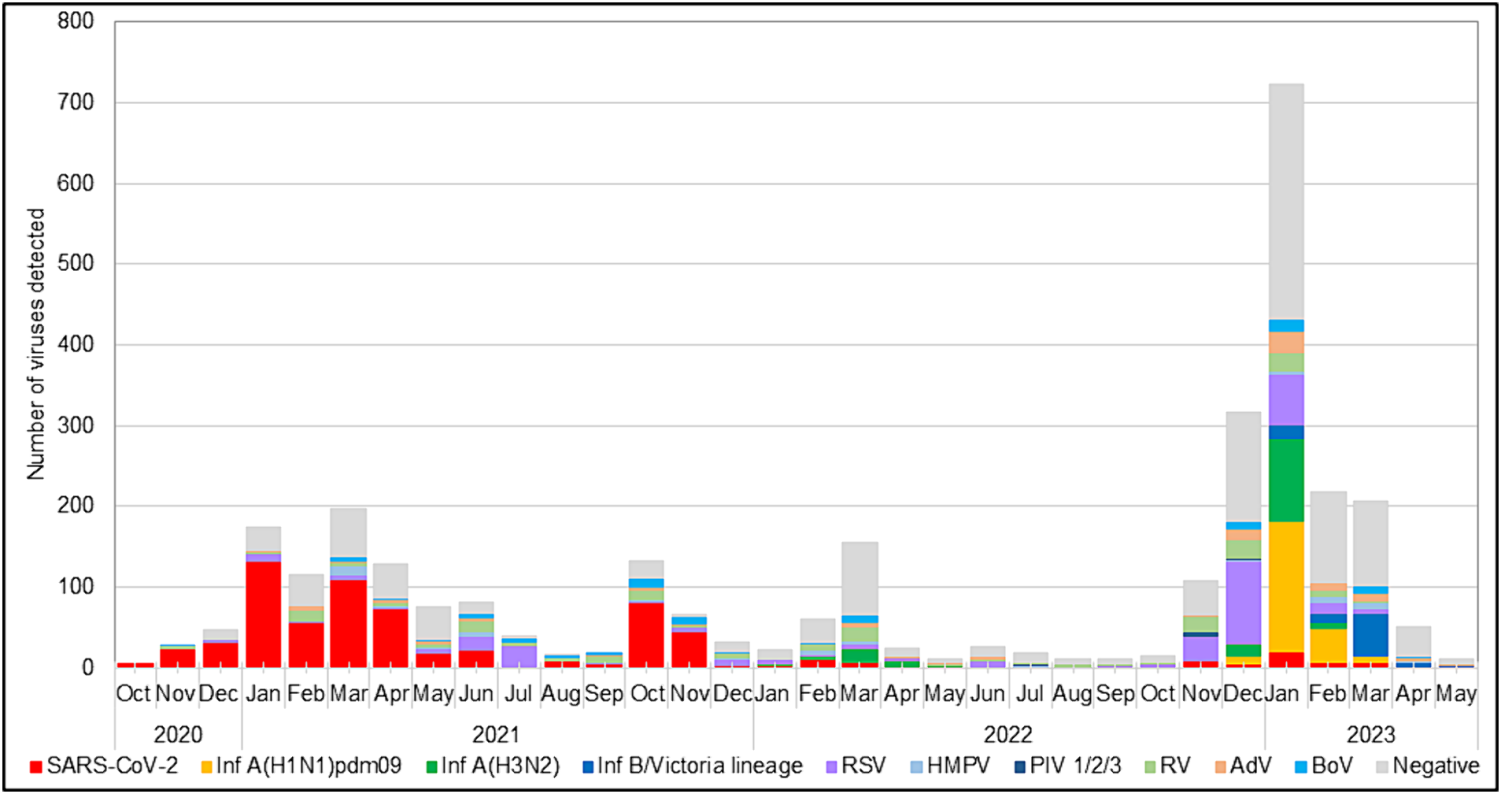
Monthly distribution of respiratory viruses detected among patients with ARI.

During the 2022–2023 season, 217 (99%) RSVs identified were subgrouped as RSV-B, and only two (1%) were RSV-A. The RSV epidemic started in October 2022 and peaked in December 2022, a month earlier than the usual peak observed during the pre-pandemic seasons (Korsun et al., 2021). RSV was mainly identified in specimens collected between November 2022 and January 2023. Throughout the study period, the highest detection rate for RSV occurred in July 2021 (75.8%, 25 cases), followed by December 2022 (33.9%, 103 cases), when the incidence rates of SARS-CoV-2 were low (3% and 1.3%, respectively; Figure 1).

### 3.3 Age and gender distribution

**Figure 2:**
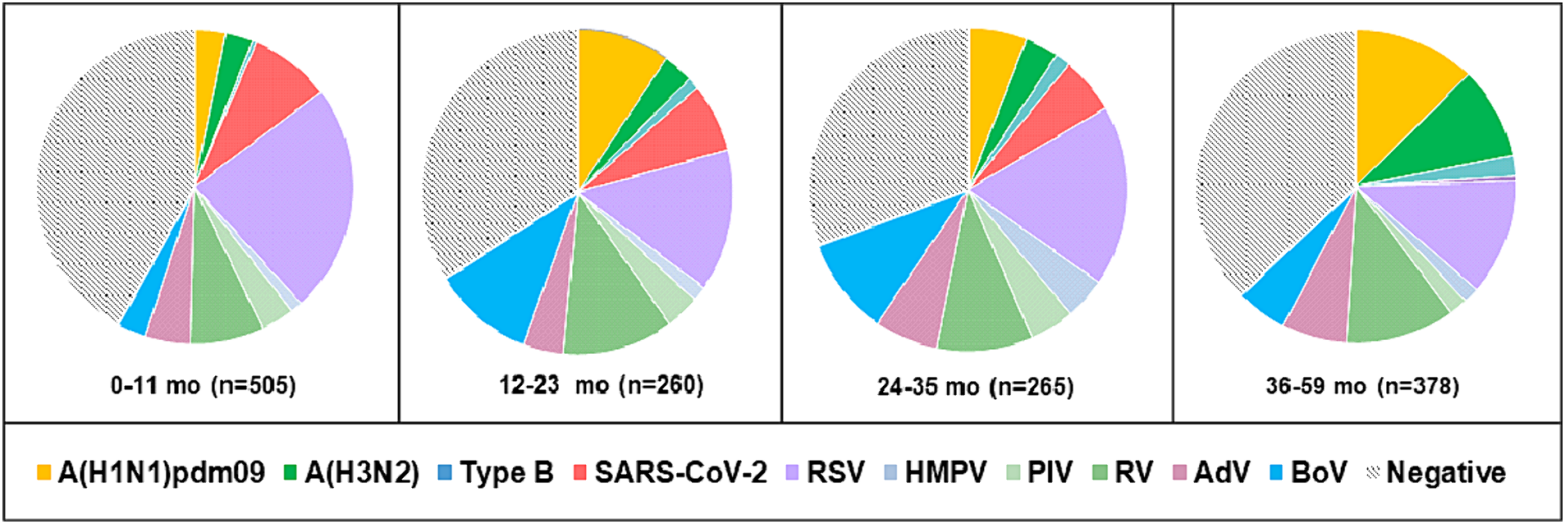
Age distribution of patients aged 0–59 months with detected respiratory viruses. Positive cases represent the sum of single infections and co-infections for each virus.

Viral respiratory infections were observed in all age groups. The proportion of positive cases in the age groups < 11 months, 11–23 months, 24–35 months 36–59 months, 5–14 years, 15–29 years, 30–64 years, and ≥ 65 years was 49.5% (250/505), 58.5% (152/260), 61.1% (162/265), 57.9% (219/378), 48% (424/883), 49.3% (67/136), 60.2% (59/98), and 96.7% (440/455), respectively. Co-infections were detected more often in patients aged 0–4 years (7.6%, 107/1,408). The highest incidence rate for SARS-CoV-2 was found in patients aged ≥ 65 years (95.6%), and for influenza viruses, the highest rate occurred in 5–14-year-olds (27.2%). Seasonal non-influenza viruses were most prevalent in the 24–35-month age group, accounting for 52.8%. Patients infected with RSV ranged in age from 1 month to 67 years, with a mean age of 4.3 ± 11.3 years and a median age of 2 years. The incidence of RSV infection was highest among the youngest age group (0–11 months, 17.7%; Figure 2) and decreased with increasing age to 1.3% in the oldest age group (≥ 65 years). A relatively high rate (6%) of RSV infection was observed in older children and adolescents (5–14 years). Children aged 0–4 years accounted for 75.5% (250/331) of the patients with confirmed RSV infection and 84.3% (198/236) of those hospitalized because of RSV infection; the highest rate of RSV co-infection (75%, 51/68) was also found in this age group (Table 2). Among the RSV-positive patients of known sex, the male-to-female ratio was 1.22 (180 males and 147 females; *p* = 0.3791), and among the RSV-positive hospitalized patients, the ratio was 1.24 (129 males and 104 females).

**Table 2:** Age distribution of patients with confirmed RSV mono-infections and co-infections.

Age group (years)	RSV positive (*n* = 331)		Mono-infections	Dual infections	Triple infections
	*n*	%			
0–4 years (*n* = 1,408)	249	17.7	199	49	2
5–14 years (*n* = 883)	53	6.0	45	8	-
15–29 years (*n* = 136)	6	4.4	5	1	-
30–64 years (*n* = 98)	3	3.1	3	0	-
≥ 65 years (*n* = 455)	6	1.3	1	5	-
Without data (67)	13	19.4	10	3	-
Total (*n* = 3,047)	331	10.9	263	66	2

### 3.4 Clinical characteristics

**Figure 3:**
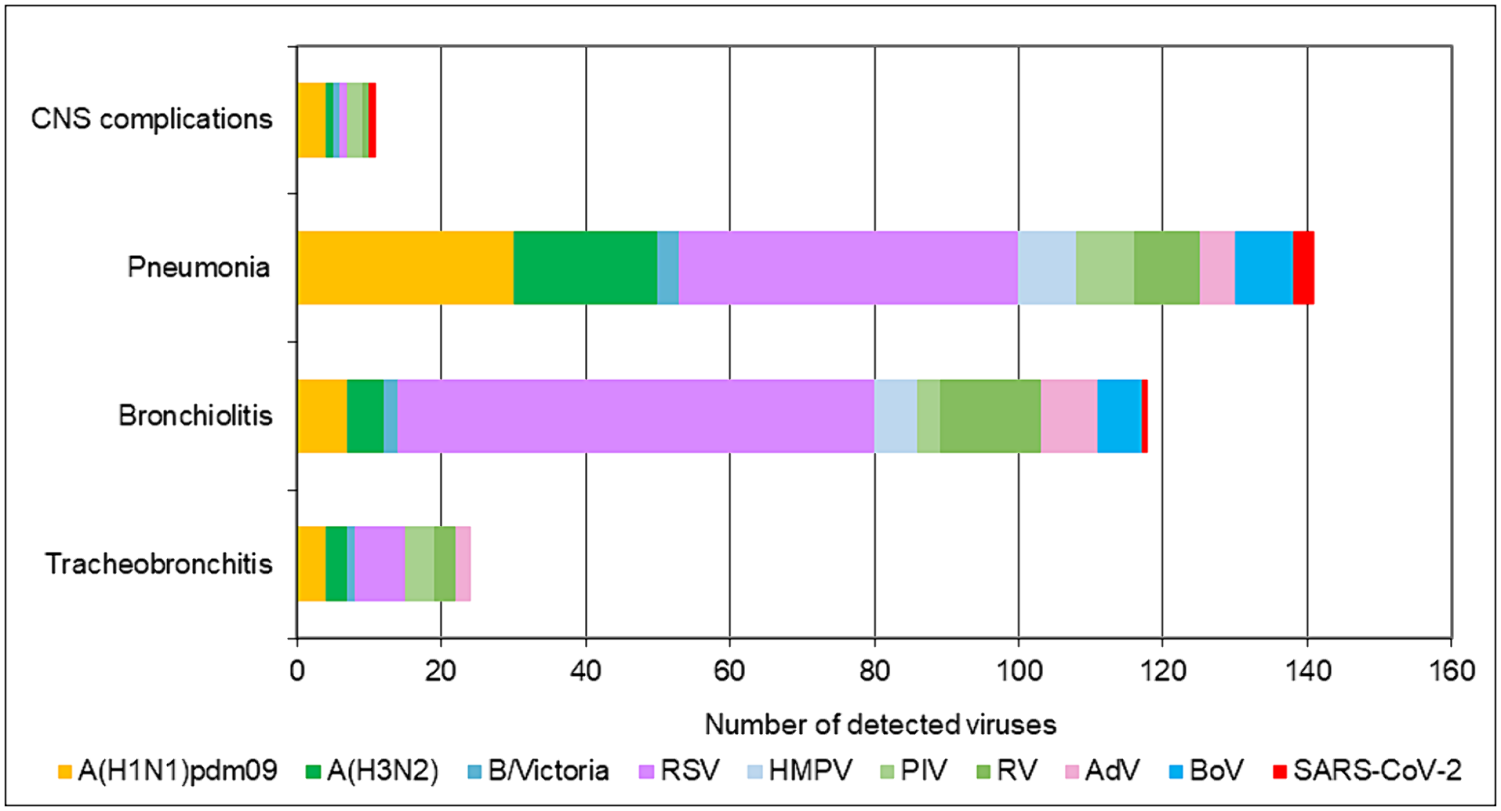
Number of respiratory viruses detected in patients with tracheobronchitis, bronchiolitis, pneumonia, and CNS complications during the 2022–2023 season.

In addition to upper respiratory tract symptoms, respiratory viruses can cause complications in the lower respiratory tract, heart, and central nervous system. We explored the participation of SARS-CoV-2, influenza viruses, and seasonal non-influenza respiratory viruses in the development of the most common complications—tracheobronchitis, bronchiolitis, pneumonia, and central nervous system involvement (febrile seizures, cerebral edema, viral meningitis, and encephalopathy). Data from the 2022–2023 season were analyzed, as they contained more complete clinical information. The proportions of influenza viruses detected in patients with tracheobronchitis, bronchiolitis, pneumonia, and neurological complications were 21.6% (8/37), 7.6% (13/170), 22.2% (53/239), and 24% (6/25), respectively; regarding seasonal non-influenza viruses, the proportions were 35.1% (13/37), 53.5% (91/170), 31% (74/239), and 12% (3/25), respectively. In SARS-CoV-2-infected patients, the proportions were 2.7%, 0.6%, 1.3%, and 4%, respectively (Figure 3). RSV was the most common virus identified in patients with bronchiolitis, accounting for 40% (68/170) of cases, and was the second most common cause of pneumonia after influenza viruses, responsible for 19.7% (47/239) of cases in the entire study population (*p* < 0.05). A total of 142 children aged <5 years were diagnosed with pneumonia, of whom 36 (25.4%) and 28 (19.7%) had confirmed RSV and influenza infections, respectively. Among the patients with lower respiratory tract complications due to RSV, 80% (72/90) were children under 2 years of age.

### 3.5 Phylogenetic analysis of RSV

**Figure 4:**
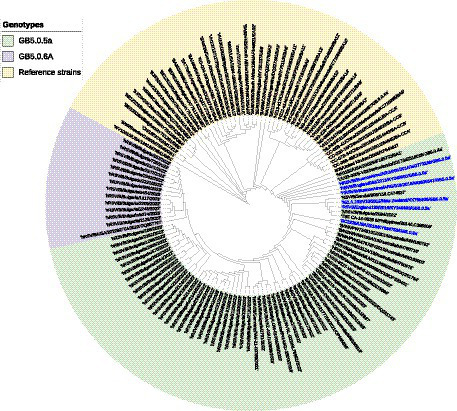
Phylogenetic analysis based on G gene nucleotide sequences of RSV-B strains. A phylogenetic tree was constructed using the maximum-likelihood method with 1,000 bootstrap iterations running within Geneious Prime software. Reference sequences were labeled with GenBank names and accession numbers, followed by country, year of isolation, and genotype. Reference RSV-B strains are indicated in yellow background. GB5.0.5a and GB5.0.6a strains detected during 2022–2023 are indicated in green and purple background, respectively. The names of the reference GB5.0.5a strains are highlighted in blue.

A phylogenetic tree based on G gene nucleotide sequences of RSV-B strains was created, including RSV-B strains detected in this study, recently circulating viruses in other countries, and representative strains of all known genotypes from GenBank. The phylogenetic analysis illustrated that the 34 sequenced Bulgarian RSV-B strains clustered together with the consensus sequence of GB5.0.5a, and the remaining 13 strains were classified into a new lineage, GB5.0.6a, within the GB5 genotype (Figure 4). The Bulgarian sequences were phylogenetically close to the sequences of RSV circulating during the same period in other European countries (the United Kingdom, Germany, Austria, Spain, Italy, and Macedonia) and the United States.

At the nucleotide level, the average patristic distance between the study GB5.0.5a strains and another GB5.0.5a strain (KY249660) was 0.0282 (±0.0021). In the remaining 13 sequences, the patristic distance with the KY249660 strain was 0.0328 (SD 0.0032), indicating that these sequences represent a new lineage of RSV-B, assigned as GB5.0.6a (Goya et al., 2020).

Next-generation sequencing identified 17 strains in which, in addition to the RSV sequence, high-quality sequence of another respiratory virus was found: SARS-CoV-2 (8 strains), influenza A(H1N1)pdm09 (3), A(H3N2) (2), B/Victoria lineage (1), and AdV (1). In strain RSV/B/Bulgaria/1305/2023, two additional sequences (SARS-CoV-2 and BoV) were identified, and in strain RSV/B/Bulgaria/2559/2022, three sequences (SARS-CoV-2, influenza virus, and BoV) were identified.

### 3.6 Amino acid polymorphisms in G and F surface proteins

Amino acid polymorphisms of the G and F surface glycoproteins reflect the evolutionary dynamics of RSV. Therefore, the deduced G and F protein amino acid sequences of the 47 Bulgarian RSV-B strains were aligned and compared with the sequence of the NH1182 strain (accession number MF185752), which is one of the first BA strains to have a complete published genome. Reference strain hRSV/B/Australia/VIC-RCH056/2019 (EPI_ISL_1653999) and eight database-derived sequences of RSV-B strains with complete genomes, detected in other countries during 2021–2023, were also included in the analysis. The study identified amino acid substitutions in the G protein at 18 positions. Among these substitutions, HVR1 (aa 67–163) contained eight, while the heparin-binding domain (aa 186–223) contained two, and HVR2 (aa 224–311) contained eight. However, no changes were observed in the cytoplasmic (aa 1–35), transmembrane (aa 36–66), or centrally conserved regions (aa 164–185; Table 3; Jiang et al., 2023).

**Table 3:** Amino acid variations identified in G and F proteins of RSV-B strains (*n* = 47) circulating in Bulgaria during the 2022–2023 season.

G protein (aa 1–315)		F protein (aa 1–574)			
Amino acid changes	Frequency (%)	Antigenic sites	Amino acid positions of antigenic sites	Amino acid changes	Frequency (%)
S100G	77	Ø	62–96; 195–227	N201S	28
S101P	100			I206M	98
T107A	98			Q209R	98
Y112H	96			S211N	96
P120L	28	I	27–45; 312–318; 378–389	F45L	100
R136T	100			S389P	96
T138S	100	II	254–277	-	
A141T	100	III	46–54; 301–311; 345–352; 367–378	-	
I200T	100	IV	422–471	-	
P216S	96	V	55–61; 146–194; 287–300	L172Q	100
S247P	100			S173L	100
D253N + CHO	28			S190N	96
K258N + CHO	96			K191R	98
V271A	98	P27	110–136	M115T	23
S277P	96			A103V	100
I281T	100			H250Y	100
T290I	98				
T312I -CHO	100				

Gain/Loss of glycosylation is represented by + CHO/-CHO.

**Figure 5:**
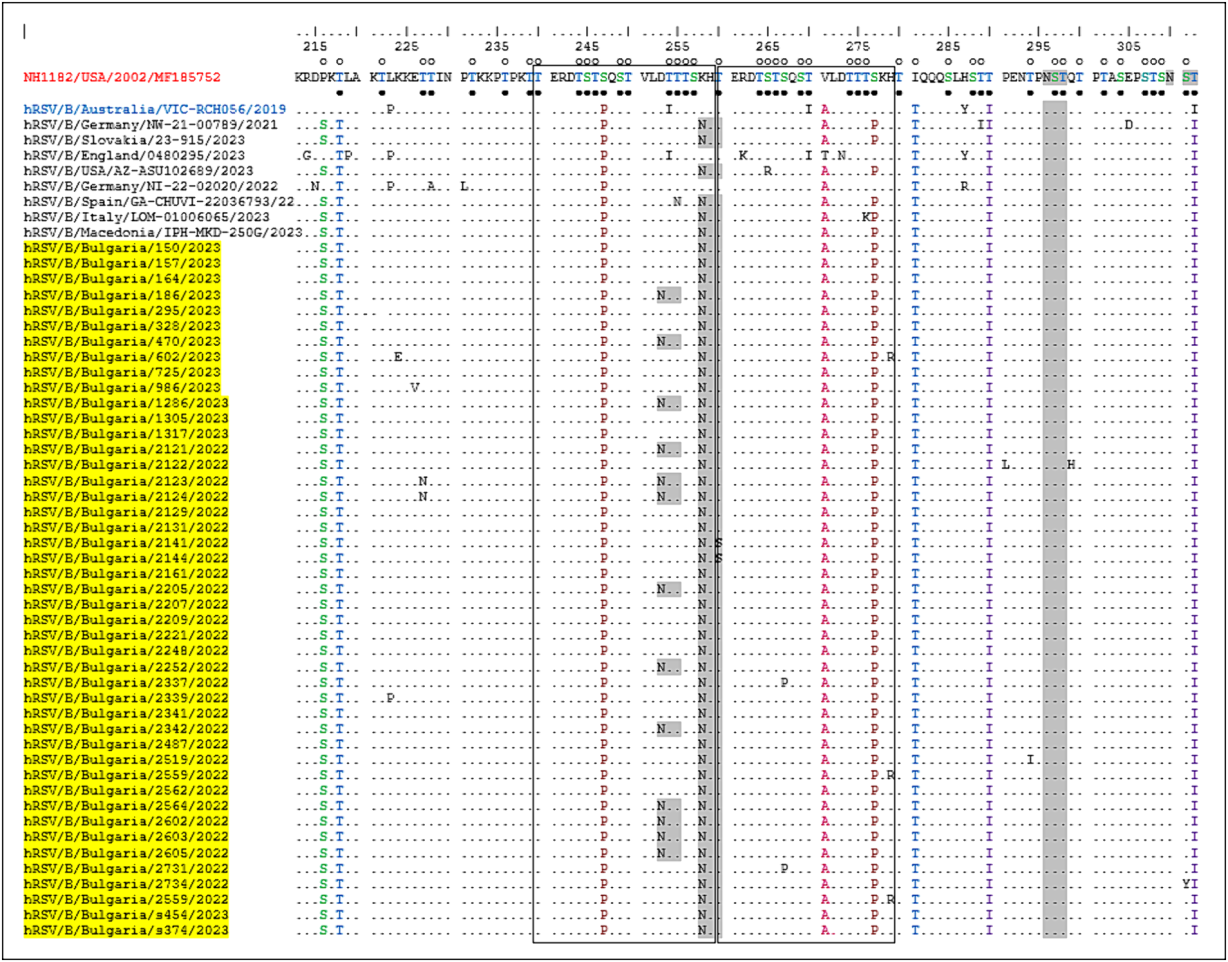
Deduced amino acid alignment of the G protein HVR2 in the RSV-B strains. Alignment is shown relative to the sequence of the prototype BA strain NH1182 (GenBank accession number MF185752). The amino acid numbers correspond to G protein positions 213–312 of strain MF185752. Identical residues are indicated by dots. Outlined rectangles represent two copies of the duplicated 20-amino acid region in the RSV-B strains. Light gray shading highlights the predicted N-glycosylation sites. Black circles indicate the predicted O-glycosylation sites of the BA prototype strain MF185752, and unfilled circles indicate the predicted O-glycosylation sites of the Bulgarian strains.

One of the unique characteristics of the BA genotypes is the presence of a 60-nucleotide duplication, leading to a duplication of 20 amino acids (TERDTSTSQSTVLDTTTSKH; aa 240–259 and 260–279) in HVR2. Consequently, protein G is extended to 312 amino acids (the stop codon in BA viruses is set to 313 amino acids). Five of the substitutions in HVR2 were observed in the Bulgarian strains detected in the 2017–2018 season (Korsun et al., 2021), and three (D253N, K258N, and S277P) were new and not present in the sequence of the reference strain hRSV/B/Australia/VIC-RCH056/2019. The substitutions V271A and S277P were located inside the duplicated region (Figure 5).

Fifteen amino acid substitutions in the G protein were conserved and represented a high frequency exceeding 90%. All identified substitutions were also found in the database-derived RSV sequences, except for the substitutions S100G, P216S, and K258N, which were present in some strains. Six N-linked glycosylation motifs were predicted in the G protein (at positions 81, 86, 230, 253, 258, and 296), three located in HVR2. The amino acid substitutions D253N and K258N led to the acquisition of novel N-linked glycosylation motifs in 28 and 96% of the strains, respectively. In contrast, substitution T312NI resulted in a loss of potential N-linked glycosylation sites in all strains. In HVR2, O-linked glycosylation was predicted in 42 serine and threonine residues with a G score ≥ 0.5, and 10 of these sites were found in the duplication region.

**Figure 6:**
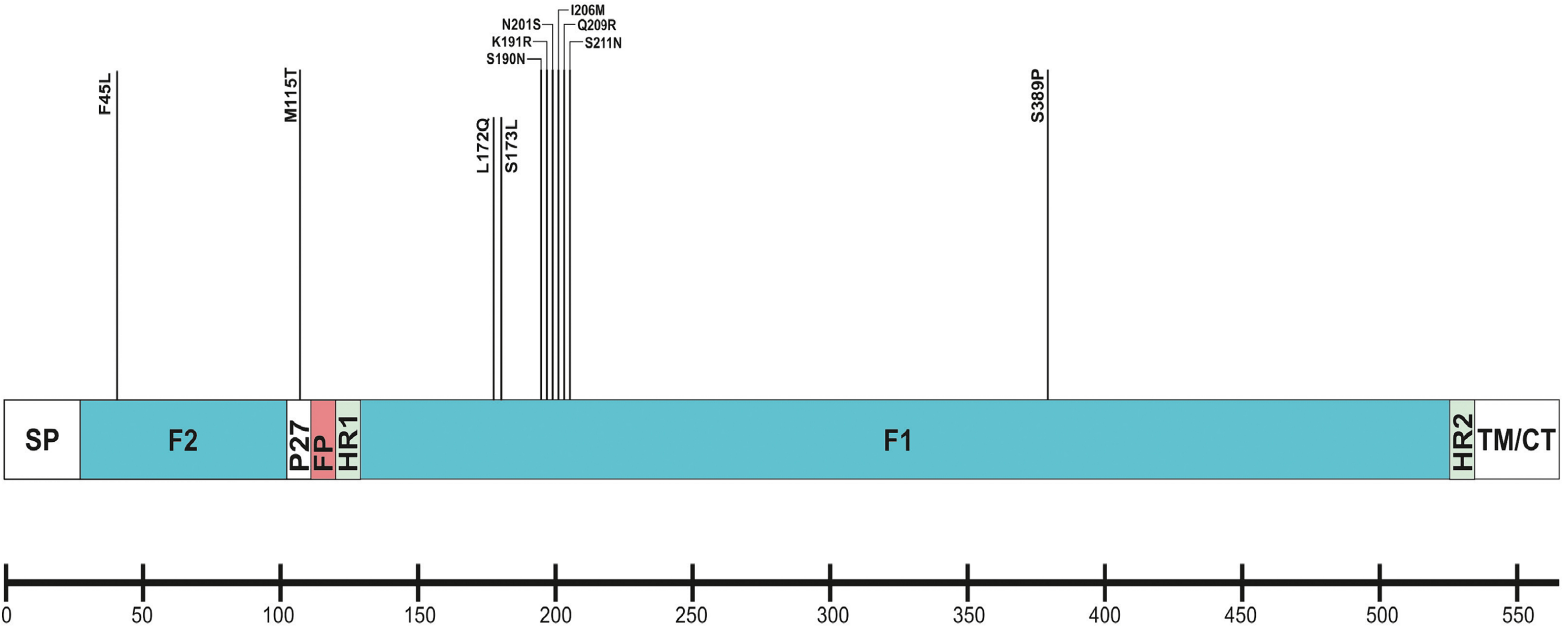
Location of amino acid substitutions identified in RSV-B F protein. Structural sites of the F protein sequence are shown: SP (signal peptide), F2 subunit (aa 24–109); p27 peptide; FP (fusion peptide); F1 subunit (aa 137–524); HR1 and HR2 (heptad repeats); and TM/CT (transmembrane/carboxy terminus).

The F protein showed variations at 13 amino acid positions: two in F2 (aa 24–109), one in the p27 segment (aa 110–136), and 10 in the F1 subunit (aa 137–524; Lu et al., 2019). No amino acid substitutions were identified in the fusion peptide (aa 27–36; Figure 6). Amino acid variations were examined in the antigenic sites Ø and I–V (Tabor et al., 2020).

No amino acid changes were identified in the major neutralizing epitopes, specifically antigenic sites II, III, and IV, found on both the pre-and post-fusion conformations of the RSV F protein. Eleven amino acid changes were detected at the remaining three antigenic sites. Nine of these had a high level of sequence conservation and were present at a frequency above 96%. Polymorphisms (N201S, I206M, Q209R, and S211N) were observed in the immunodominant Ø epitope, which was unique to the pre-fusion conformation of the F protein (McLellan, 2015). The substitutions L172Q, S173L, K191R, and K191R were located at site V, F45L, and S389P at site I, and M115T at the p27 segment—an internal peptide released after cleavage of the two subunits F1 and F2. Only two substitutions (A103V and H250Y) were located outside the antigenic regions (Table 3). Except for S190N, S211N, and S389P, all analyzed database-derived RSV sequences contained the remaining substitutions. Six N-linked glycosylation motifs were predicted in the F protein (at aa positions 27, 70, 116, 120, 126, and 500). Three glycosylation sites (N116, N120, and N126) were located within the p27 segment.

### 3.7 Complete SH, N, P, M, M2-1, M2-2, and L protein analysis

Single amino acid substitutions were identified in other structural proteins: T49I and N64D in the small hydrophobic surface protein (SH; 65 aa in length); H216Y and A372T in the nucleoprotein (N) (391 aa); G229D in the phosphoprotein (P) (241 aa); V181I in the M2-1 (195 aa) and R41H in the M2-2 (90 aa) transcriptional regulators. No amino acid variations were identified in the matrix (M) protein (256 aa). The large (L) protein, consisting of 2,165 amino acids organized into five domains: RdRp domain, capping domain (Cap), connector domain (CD), methyltransferase domain (MT), and carboxy-terminal domain (CTD), harbored 11 amino acid variations at positions 56, 570, 715, 1,479, 1,712, 1,716, 1,723, 1,736, 1,759, 1,787, and 2,108.

## 4 Discussion

This study investigated the circulation patterns of RSV and other respiratory viruses among patients presenting with ARI symptoms in Bulgaria during the COVID-19 pandemic, with an emphasis on the first season after COVID-19 restrictions were lifted. The genetic characteristics of the RSV responsible for the 2022–2023 outbreak were also explored. Extensive non-pharmaceutical preventive interventions targeting SARS-CoV-2 have had a tremendous impact on viral respiratory infection epidemiology, such as the low prevalence and diminished genetic diversity of circulating viruses and the monthly and age distribution of cases (Garg et al., 2022; Chow et al., 2023). Viral interference, the behavior of individuals, and societal and health system factors may have also contributed to the significant reduction in respiratory virus circulation during the pandemic (Abu-Raya et al., 2023). During the first two winters, the activity of RSV and other seasonal respiratory viruses plummeted abruptly worldwide, including in Bulgaria (Hönemann et al., 2023). An unusual summer spike in RSV-associated activity was observed in 2021 during the first relaxation of COVID-19-related public health measures. The low RSV exposure during the winter of 2020–2021 probably resulted in a significant decline in population RSV-specific immunity and an atypical inter-seasonal resurgence of RSV infections (Reicherz et al., 2022; Bardsley et al., 2023). In the fall of 2022, with the complete withdrawal of containment measures, RSV returned to the seasonally high levels of activity observed in the preceding years. The sudden upsurge in RSV cases in the summer of 2021 and autumn of 2022, as well as the earlier start of the 2022–2023 epidemic, has been registered in many countries (Redlberger-Fritz et al., 2021; Goya et al., 2023; Hönemann et al., 2023; Munkstrup et al., 2023). The detection rate of RSV in the 2022–2023 outbreak was 13.7% vs. 3.2% and 6.6% during the previous two seasons, respectively (*p* < 0.05). Among all the studied respiratory viruses, RSV was the most frequently identified etiologic agent of ARIs during the 2022–2023 season and the second most commonly detected respiratory virus after SARS-CoV-2 during the previous two seasons. Temporal and geographic variations exist in the incidence of RSV infection, depending on the study population, detection methods, climate, and other factors (Gimferrer et al., 2019; Valley-Omar et al., 2022; Bimouhen et al., 2023; Kim et al., 2023). During the COVID-19 pandemic, the intensity of the applied anti-epidemic measures has played a crucial role in determining the spread of seasonal respiratory viruses. In this study, RSV was co-detected with other respiratory viruses in 68 (20.5%) patients, including SARS-CoV-2 in 14 patients. A high rate (23.3%, 55/236) of co-infections was found among hospitalized RSV-positive patients. The proportion of RSV co-infections varied between studies (Trento et al., 2010; Holmes, 2013; Gimferrer et al., 2019), and no co-infections with other respiratory viruses were detected in a study conducted in Washington, United Kingdom (Goya et al., 2023). Currently, there is no conclusive evidence linking the presence of co-infections with disease severity (Goka et al., 2014).

The strong dominance of RSV-B during the 2022–2023 epidemic can be explained by the low transmission of RSV in the previous two seasons, which could have caused a genetic bottleneck, resulting in reduced viral diversity (Chow et al., 2023). Significant declines in RSV genetic diversity during the implementation of COVID-19-related restrictions have also been observed in other countries (Eden et al., 2022). Our findings were consistent with reports from other European countries, where the 2022–2023 outbreak was also driven by RSV-B (Redlberger-Fritz et al., 2021; Munkstrup et al., 2023; Pierangeli et al., 2023). During the period preceding the pandemic, year-to-year fluctuations in the incidences of RSV-A and RSV-B were observed in Bulgaria (Korsun et al., 2021). Herd immunity against the RSV subgroup, which was dominant in the country in the preceding year, is likely the reason for the dominance of other subgroups in the following season. A consistent shift in the predominance of RSV-A to RSV-B at different time intervals (1–3 years) has been reported worldwide (Hall, 2001; Gimferrer et al., 2019).

During the study period, RSV affected all age groups across the population, with much more frequent involvement in children aged < 5 years, especially those under 2 years. In line with many reports, the rate of RSV infection was highest in the youngest age group (up to 1 year) and decreased with increasing age due to the development of immunity after repeated infections (Jepsen et al., 2018; Jallow et al., 2023). Administration of the recently approved Abrysvo vaccine during pregnancy could protect infants against LRTI from birth to 6 months of age (EMA; Saso and Kampmann, 2016). According to the literature, the burden of RSV infection is high in older adults who are at risk of severe infection (Savic et al., 2023). In contrast to a previous study (Nguyen-Van-Tam et al., 2022), the study older population aged ≥65 years was minimally affected by RSV infection (4.66% vs. 1.3%, respectively). For this age group, licensed vaccines (Arexvy and Abrysvo) have already been designed to protect against LRTI (such as bronchitis and pneumonia) caused by RSV [European Medicine Agency (EMA), 2023a,b]. Our previous study showed that RSV infections follow a seasonal pattern, occurring predominantly in winter and early spring, with very few cases occurring during the summer (Korsun et al., 2021). During the 2022–2023 season, RSV was in circulation from November to late March, with peak activity in December 2022, which is consistent with data from other European countries (Jepsen et al., 2018; Gimferrer et al., 2019; Pierangeli et al., 2023). From 2020 to 2021, the typical seasonality of RSV was disrupted because of the applied anti-COVID-19 measures. Information on the monthly distribution of RSV infections is important for the implementation of prophylactic measures, including vaccines and monoclonal antibodies, among high-risk populations and for strengthening infection control to prevent nosocomial infections.

Similar to the pre-pandemic seasons, respiratory viruses were more frequently detected among inpatients (63.5%) than outpatients (49.4%; *p* < 0.05; Korsun et al., 2021). RSV was the major pathogen associated with hospitalization in children, and 59.8% (198/331) of all RSV cases occurred in hospitalized children aged < 5 years. RSV is well known as the leading etiological agent of LRTI in the pediatric population and the predominant cause of bronchiolitis and pneumonia (Hall, 2001). During the 2022–2023 season, RSV was the most common cause of bronchiolitis (40%) and pneumonia (25.4%) in children aged < 5 years. RSV-associated cases of LRTI were observed more frequently in children younger than 2 years of age, indicating a higher susceptibility of this age group to RSV infection. According to a previous report, boys are more likely to develop severe disease, and male infants are twice as likely to be hospitalized than female infants (Hall, 2004). In our study, no statistically significant differences were found in the incidence and hospitalization rates among males and females < 5 years old with confirmed RSV infection.

Whole-genome sequencing and phylogenetic analysis of 47 RSV-B sequences indicated that the GB5.0.5a and GB5.0.6a genotypes (both equivalent to the BA strains) were responsible for the 2022–2023 outbreak. The 2022–2023 RSV-B surge was also driven by GB5.0.5a in the United States (Adams et al., 2023), Austria (Redlberger-Fritz et al., 2021), Italy (Tramuto et al., 2023), and other countries (Jallow et al., 2023). Molecular analysis of RSV G genes revealed the presence of a 60-nucleotide duplication in HVR2, which is a landmark characteristic of BA strains initially described in Buenos Aires, Argentina, in 1999. Following its appearance, the BA genotype rapidly evolved and displayed significant diversification into at least 14 new genotypes (Ábrego et al., 2017; Lee et al., 2021; Kim et al., 2023). Since 2006, RSV-B strains harboring this partial duplication have become globally predominant and have completely replaced all previously circulating RSV-B genotypes (Trento et al., 2010). The worldwide spread of RSV-B strains carrying this 60-nucleotide duplication indicates that this unique insertion may enhance viral attachment to host cells and improve fitness, thereby facilitating transmission (Hotard et al., 2015).

Surface glycoproteins G and F are under selective immune pressure and undergo constant evolution. Therefore, they were subjected to molecular analysis. In Bulgarian RSV-B, amino acid substitutions were identified at 18 G protein positions, of which 16 were located in HVR1/2 and six N-linked glycosylation motifs, including two new ones, compared to the NH1182 strain discovered 20 years ago in the early years after the appearance of the BA genotype. Duplication of 20 amino acids in the RSV-B strain resulted in additional amino acid substitutions in HVR2. Some substitutions identified in the G protein (S247P, V271A, I281T, T290I, and T312I) have been described in other European countries and elsewhere (Kim et al., 2023; Tramuto et al., 2023). The RSV F protein is a vaccine antigen and a target of the monoclonal antibody product palivizumab (Synagis), which is administered as a passive immunoprophylaxis to high-risk infants, as well as several other monoclonal antibodies and small molecules that are under clinical development (Kopera et al., 2023). Key variations in the F protein antigenic sites could affect the antigenicity and susceptibility of RSV to prophylactic and therapeutic agents targeting these regions. In our study, the F protein showed 13 substitutions and six N-linked glycosylation sites, confirming its lower genetic variability than the G protein (Sun et al., 2022). No amino acid variations were found at antigenic sites II (target of palivizumab and motavizumab) or IV (target of 101F and MAb19; Lu et al., 2019). More variation was found in the antigenic sites Ø (target of nirsevimab) and V (target of suptavumab). This variability is likely a result of neutralization escape, given that these regions prompt the production of antibodies with high neutralizing activity (Ruckwardt et al., 2019; Tabor et al., 2020; Adhikari et al., 2022; Sun et al., 2022). The antigenic site Ø is located at the apex of the pre-fusion trimer and forms much of the variability of the F protein (~25%; McLellan et al., 2013). The variations L172Q, S173L, S190N, and K191R were located at site V (aa 148–194; target of the monoclonal antibody AM14), and the substitution F45L was located at site I (aa 27–45; target of human antibody MPE8; Hause et al., 2017). The substitutions A103V, L172Q, S173L, K191R, I206M, and Q209R have also been reported in other recent studies (Lu et al., 2019; Adhikari et al., 2022; Chen et al., 2022; Sun et al., 2022). The N-linked glycosylation sites of the F protein were relatively conserved, whereas those of the G protein were more variable. The acquisition or removal of N-linked glycosylation can affect viral antigenicity and facilitate immune evasion (Feng et al., 2022). The continuous accumulation of amino acid changes and extensive glycosylation of RSV G and F proteins with N-and O-linked sugars allow viruses to escape neutralization by pre-existing antibodies. Consequently, the continued emergence of genetically altered RSV has enabled this pathogen to cause repeated infections in the same individual and annual epidemics. Full-length sequencing of other structural proteins showed a high degree of similarity with the reference strain. In agreement with a previous study, a small number of amino acid changes were identified in the internal proteins (Jallow et al., 2023). The L56I, K570R, I715V, R1759K, and A1787E substitutions were located in the conserved enzymatic regions RdRp, Cap, and MT of L protein, which are potential targets for inhibitor development (Gilman et al., 2019). A recent study in Austria reported the L protein variations K570R, V1479A, and R1759K, which were also observed in Bulgarian strains (Redlberger-Fritz et al., 2021).

It is worth noting that this study had a few limitations. In the first two seasons of the COVID-19 pandemic, the number of clinical samples examined was notably smaller than that in the 2022–2023 season. This is because the national ARI surveillance system was disrupted during this period, as resources and specialists were redirected toward COVID-19 diagnosis, treatment, and contact tracing. A significant portion of the National Laboratory’s “Influenza and ARI” work involved testing for SARS-CoV-2. The reporting forms for clinical samples were often incomplete and lacked the necessary clinical information. This made it impossible to perform an extensive epidemiological and clinical analysis of respiratory infections other than SARS-CoV-2 during the acute phase of the pandemic. In addition, due to the limited detection of RSV-A, we did not sequence viruses in this subgroup and analyze their genetic characteristics. Finally, as the objectives of the study focused on RSV infection, other respiratory infections caused by RVs, AdVs, PIVs, and BoVs were not analyzed in detail. Despite these limitations, our study provides a comprehensive overview of RSV circulation patterns during the COVID-19 pandemic. Policymakers can utilize this information to devise effective strategies for controlling the transmission of RSV and preventing future epidemics. We identified complete genome sequences of one or more respiratory viruses in addition to the RSV sequence in some strains, a unique finding not present in other publications.

This study found a high incidence of viral respiratory infections, particularly RSV, during the first season after anti-COVID-19 restrictions were lifted. The high activity of RSV is likely a result of diminished population immunity and the accumulation of vulnerable individuals, particularly children, due to prolonged low exposure to natural infections. We identified RSV as the primary cause of severe respiratory illnesses in young children. This study emphasizes the need for ongoing local and global surveillance of this pathogen. Phylogenetic and molecular analyses of RSV play crucial roles in identifying new epidemic strains, tracking the evolutionary and epidemiological patterns of viruses, and evaluating the impact of genetic variation on the transmissibility, virulence, and effectiveness of preventative vaccines and medications.

## Data availability statement

The datasets presented in this study can be found in online repositories. The names of the repository/repositories and accession number(s) can be found in the article/Supplementary material.

## Ethics statement

The studies involving humans were approved by Institutional Review Board and Ethics Committee of the NCIPD (IRB Number 00006384). The studies were conducted in accordance with the local legislation and institutional requirements. Written informed consent for participation in this study was provided by the participants’ legal guardians/next of kin.

## Author contributions

NK: Conceptualization, Data curation, Formal analysis, Investigation, Visualization, Writing – original draft, Methodology. IT: Data curation, Formal analysis, Investigation, Methodology, Visualization, Validation, Writing – review & editing. IM: Data curation, Formal analysis, Investigation, Methodology, Writing – review & editing. IA: Methodology, Resources, Writing – review & editing. IU: Data curation, Formal analysis, Writing – review & editing. II: Data curation, Formal analysis, Writing – review & editing. PV: Data curation, Formal analysis, Writing – review & editing. TT: Data curation, Formal analysis, Writing – review & editing. IC: Formal analysis, Supervision, Writing – review & editing, Data curation.

## Footnotes

^1^ https://ecdc.europa.eu/en/infectious-diseases-public-health/surveillance-and-disease-data/eu-case-definitions

^2^ https://support-docs.illumina.com/LP/Illumina_RNA_Prep_Checklist/Content/LP/Illumina_RNA/RNA-Prep/Checklist.htm

^3^ https://www.geneious.com/

^4^ https://itol.embl.de/

^5^ https://services.healthtech.dtu.dk/service.php?NetNGlyc-1.0
