# Supplementary material for: Resurgence of respiratory syncytial virus with dominance of RSV-B during the 2022–2023 season
Source: Front Microbiol. 2024 Apr 2;15:1376389. doi: 10.3389/fmicb.2024.1376389 (PMC11019023; doi:10.3389/fmicb.2024.1376389)
Supplement: Supplementary file 1 [file Table_1.doc]

Supplementary Table 1. Primers/probes used in this study and thermocycling conditions

| Assays | Target gene | Primer/probe sequence (5'–3') | Final conc (nM) | Thermo  cycling conditions |
| --- | --- | --- | --- | --- |
| *Real time*  *RT-PCR for*  RSV detection | M | F: GGC AAA TAT GGA AAC ATA CGT GAA  R: TCT TTT TCT AGG ACA TTG TAY TGA ACA G  P: FAM-CTG TGT ATG TGG AGC CTT CGT GAA GCT-BHQ1 | 500  250  50 | RT at 45^0^C for 10 min, 94^0^C for 10 min, 45 cycles (94^0^C for 30 s, 60^0^C for 1 min) |
| PIV-1 detection | HN | F: AGT TGT CAA TGT CTT AAT TCG TAT CAA T  R: TCG GCA CCT AAG TAA TTT TGA GTT  P: FAM-ATA GGC CAA AGA T(BHQ1)TG TTG TCG AGA CTA TTC CAA | 500  500  50 |
| PIV-2 detection | HN | F: GCA TTT CCA ATC TAC AGG ACT ATG A  R: ACC TCC TGG TAT AGC AGT GAC TGA AC  P: FAM-CCA TTT ACC T(BHQ1)AA GTG ATG GAA TCA ATC GCA AA | 750  750  50 |
| PIV-3 detection | HN | F: TGG YTC AAT CTC AAC AAC AAG ATT TAA G  R: TAC CCG AGA AAT ATT ATT TTG CC  P: FAM-CCC RTC TGT(BHQ1)TGG ACC AGG GAT ATA CTA CAA A | 750  500  200 |
| hMPV detection | F | F: CAA GTG TGA CAT TGC TGA YCT RAA  R: ACT GCC GCA CAA CAT TTA GRA A  P: FAM-TGG CYG TYA GCT TCA GTC AAT TCA ACA GA-BHQ1 | 600  600  100 |
| RV detection | 5’NCR | F: CP**A** GCC **T**GC GTG GC  R: GAA ACA CGG ACA CCC AAA GTA  P: FAM-TCC TCC GGC CCC TGA ATG YGG C- BHQ1 | 1000  1000  100 |
| AdV detection  BoV detection | Hexon  NS1 | F: GCC CCA GTG GTC TTA CAT GCA CAT C  R: GCC ACG GTG GGG TTT CTA AAC TT  P: FAM-TGC ACC AGA CCC GGG CTC AGG TAC TCC GA-BHQ1  F: TGC AGA CAA CGC YTA GTT GTT T  R: CTG TCC CGC CCA AGA TAC A  P: FAM-CCA GGA TTG GGT GGA ACC TGC AAA--BHQ1 | 500  500  100  500  500  100 |
| RSV-A | F | AFF: CTG TGA TAG ART TCC AAC AAA AGA ACA  AFR: AGT TAC ACC TGC ATT AAC ACT AAA TTC C  P: MGB**-**CAG ACT ACT AGA GAT TAC C-BHQ1 | 900  900  100 | RT at 48°C for 30 min, 95°C for 10 min, 45 cycles (95°C for 15 s, 60°C for 1 min)^14^ |
| RSV-B | N | BNF: GGC TCC AGA ATA TAG GCA TGA TTC  BNR: TGG TTA TTA CAA GAG CAG CTA TAC ACA GT  P: VIC**-**TAT CAT CCC ACA GTC TG-BHQ1 | 900  900  100 |

**Abbreviations:** NCR, noncoding region; F, forward primer; R, reverse primer; P, probe; FAM, 6-carboxyfluorescein; BHQ, black hole quencher; MGB, minor groove binder. Underlining and boldface indicate a locked nucleic acid.
