# Supplementary material for: Resurgence of respiratory syncytial virus with dominance of RSV-B during the 2022–2023 season
Source: Front Microbiol. 2024 Apr 2;15:1376389. doi: 10.3389/fmicb.2024.1376389 (PMC11019023; doi:10.3389/fmicb.2024.1376389)
Supplement: Supplementary file 2 [file Table_2.docx]

**Supplementary Table 2**. GISAID virus/sequence identification/accession numbers of Bulgarian RSV-B strains analyzed in this study

| **RSV-B strains** | **Accession number** |
| --- | --- |
| hRSV/B/Bulgaria/150/2023 | EPI_ISL_18447485 |
| hRSV/B/Bulgaria/157/2023 | EPI_ISL_18447482 |
| hRSV/B/Bulgaria/164/2023 | EPI_ISL_18447483 |
| hRSV/B/Bulgaria/186/2023 | EPI_ISL_18447484 |
| hRSV/B/Bulgaria/295/2023 | EPI_ISL_18447486 |
| hRSV/B/Bulgaria/328/2023 | EPI_ISL_18447487 |
| hRSV/B/Bulgaria/470/2023 | EPI_ISL_18447489 |
| hRSV/B/Bulgaria/602/2023 | EPI_ISL_18447490 |
| hRSV/B/Bulgaria/725/2023 | EPI_ISL_18447488 |
| hRSV/B/Bulgaria/986/2023 | EPI_ISL_18447491 |
| hRSV/B/Bulgaria/1286/2023 | EPI_ISL_18447492 |
| hRSV/B/Bulgaria/1305/2023 | EPI_ISL_18447493 |
| hRSV/B/Bulgaria/1317/2023 | EPI_ISL_18447494 |
| hRSV/B/Bulgaria/2121/2022 | EPI_ISL_18447451 |
| hRSV/B/Bulgaria/2122/2022 | EPI_ISL_18447452 |
| hRSV/B/Bulgaria/2123/2022 | EPI_ISL_18447453 |
| hRSV/B/Bulgaria/2124/2022 | EPI_ISL_18447454 |
| hRSV/B/Bulgaria/2129/2022 | EPI_ISL_18447456 |
| hRSV/B/Bulgaria/2131/2022 | EPI_ISL_18447457 |
| hRSV/B/Bulgaria/2141/2022 | EPI_ISL_18447458 |
| hRSV/B/Bulgaria/2144/2022 | EPI_ISL_18447455 |
| hRSV/B/Bulgaria/2161/2022 | EPI_ISL_18447459 |
| hRSV/B/Bulgaria/2162/2022 | EPI_ISL_18447460 |
| hRSV/B/Bulgaria/2205/2022 | EPI_ISL_18447461 |
| hRSV/B/Bulgaria/2207/2022 | EPI_ISL_18447462 |
| hRSV/B/Bulgaria/2209/2022 | EPI_ISL_18447463 |
| hRSV/B/Bulgaria/2221/2022 | EPI_ISL_18447466 |
| hRSV/B/Bulgaria/2248/2022 | EPI_ISL_18447467 |
| hRSV/B/Bulgaria/2252/2022 | EPI_ISL_18447464 |
| hRSV/B/Bulgaria/2337/2022 | EPI_ISL_18447465 |
| hRSV/B/Bulgaria/2339/2022 | EPI_ISL_18447469 |
| hRSV/B/Bulgaria/2341/2022 | EPI_ISL_18447470 |
| hRSV/B/Bulgaria/2342/2022 | EPI_ISL_18447471 |
| hRSV/B/Bulgaria/2487/2022 | EPI_ISL_18447472 |
| hRSV/B/Bulgaria/2519/2022 | EPI_ISL_18447468 |
| hRSV/B/Bulgaria/2559/2022 | EPI_ISL_18447473 |
| hRSV/B/Bulgaria/2562/2022 | EPI_ISL_18447474 |
| hRSV/B/Bulgaria/2564/2022 | EPI_ISL_18447475 |
| hRSV/B/Bulgaria/2594/2022 | EPI_ISL_18447476 |
| hRSV/B/Bulgaria/2602/2022 | EPI_ISL_18447477 |
| hRSV/B/Bulgaria/2603/2022 | EPI_ISL_18447478 |
| hRSV/B/Bulgaria/2605/2022 | EPI_ISL_18447479 |
| hRSV/A/Bulgaria/2715/2022 | EPI_ISL_18447450 |
| hRSV/B/Bulgaria/2731/2022 | EPI_ISL_18447480 |
| hRSV/B/Bulgaria/2734/2022 | EPI_ISL_18447498 |
| hRSV/B/Bulgaria/s121/2023 | EPI_ISL_18447481 |
| hRSV/B/Bulgaria/s374/2023 | EPI_ISL_18447495 |
| hRSV/B/Bulgaria/s454/2023 | EPI_ISL_18447496 |
